# *Clostridium perfringens* strains from bovine enterotoxemia cases are not superior in *in vitro* production of alpha toxin, perfringolysin O and proteolytic enzymes

**DOI:** 10.1186/1746-6148-10-32

**Published:** 2014-01-30

**Authors:** Evy Goossens, Stefanie Verherstraeten, Leen Timbermont, Bonnie R Valgaeren, Bart Pardon, Freddy Haesebrouck, Richard Ducatelle, Piet R Deprez, Filip Van Immerseel

**Affiliations:** 1 Department of Pathology, Bacteriology and Avian Diseases, Faculty of Veterinary Medicine, Ghent University, Salisburylaan 133, B-9820 Merelbeke, Belgium; 2 Department of Internal Medicine and Clinical Biology of Large Animals, Faculty of Veterinary Medicine, Ghent University, Salisburylaan 133, B-9820 Merelbeke, Belgium

## Abstract

**Background:**

Bovine enterotoxemia is a major cause of mortality in veal calves. Predominantly veal calves of beef cattle breeds are affected and losses due to enterotoxemia may account for up to 20% of total mortality. *Clostridium perfringens* type A is considered to be the causative agent. Recently, alpha toxin and perfringolysin O have been proposed to play an essential role in the development of disease. However, other potential virulence factors also may play a role in the pathogenesis of bovine enterotoxemia. The aim of this study was to evaluate whether strains originating from bovine enterotoxemia cases were superior in *in vitro* production of virulence factors (alpha toxin, perfringolysin O, mucinase, collagenase) that are potentially involved in enterotoxemia. To approach this, a collection of strains originating from enterotoxemia cases was compared to bovine strains isolated from healthy animals and to strains isolated from other animal species.

**Results:**

Strains originating from bovine enterotoxemia cases produced variable levels of alpha toxin and perfringolysin O that were not significantly different from levels produced by strains isolated from healthy calves and other animal species. All tested strains exhibited similar mucinolytic activity independent of the isolation source. A high variability in collagenase activity between strains could be observed, and no higher collagenase levels were produced *in vitro* by strains isolated from enterotoxemia cases.

**Conclusions:**

Bovine enterotoxemia strains do not produce higher levels of alpha toxin, perfringolysin O, mucinase and collagenase, as compared to strains derived from healthy calves and other animal species *in vitro*.

## Background

Bovine enterotoxemia caused by *Clostridium perfringens*, is a sudden death syndrome with necro-hemorrhagic lesions in the small intestine, which mainly affects suckling calves and veal calves [1,2]. In veal calves, predominantly beef cattle breeds are affected. The syndrome accounts for approximately 20% of the mortalities in these calves, compared to 4% in dairy and mixed breed veal calves [2,3]. *Clostridium perfringens* is an anaerobic gram-positive spore-forming bacterium, which is a commensal of the gastrointestinal tract of both humans and animals, and is also ubiquitous in soil and sewage [2,4,5]. Strains of *C. perfringens* are classified into five toxinotypes (A-E) based on the presence of four major toxin genes (alpha, beta, iota and epsilon) [6]. Mainly strains of type A are recovered from enterotoxemia cases but also from the intestine of healthy cattle and other warm-blooded animals as well as from the environment [6]. *C. perfringens* type A has the ability to produce numerous extracellular toxins and enzymes, of which alpha toxin is the most toxic [7]. Recently alpha toxin, a phospholipase C, and perfringolysin O, a pore forming cytolysin, have been proposed as essential factors for induction of enterotoxemia [8]. In addition to these toxins, other potential virulence factors might have a role in the pathology of enterotoxemia. Possible virulence traits can be proteolytic factors that degrade the protective mucus layer and extracellular matrix components or intra-species inhibitory antibacterial substances that confer a selective advantage to the producing strain, as suggested for necrotic enteritis strains in broilers [9].

It is hitherto unclear whether the *C. perfringens* type A strains isolated from enterotoxemia cases are more virulent than other type A strains. In a calf intestinal loop model, it has been shown that *C. perfringens* strains isolated from healthy and enterotoxemic cattle as well as from other host species are all capable of inducing necro-hemorrhagic intestinal lesions [10].

The purpose of this study was to analyze the expression of virulence factors that are potentially involved in enterotoxemia. To approach this, the alpha toxin and perfringolysin O production, the mucinolytic and gelatinolytic activity as well as the intra-species inhibitory activity of a collection of strains originating from enterotoxemia cases was compared to bovine strains isolated from healthy animals and to strains isolated from other animal species.

## Results

### *Clostridium perfringens* strains from enterotoxemia cases are not superior alpha toxin and perfringolysin O producers

**Figure 1:**
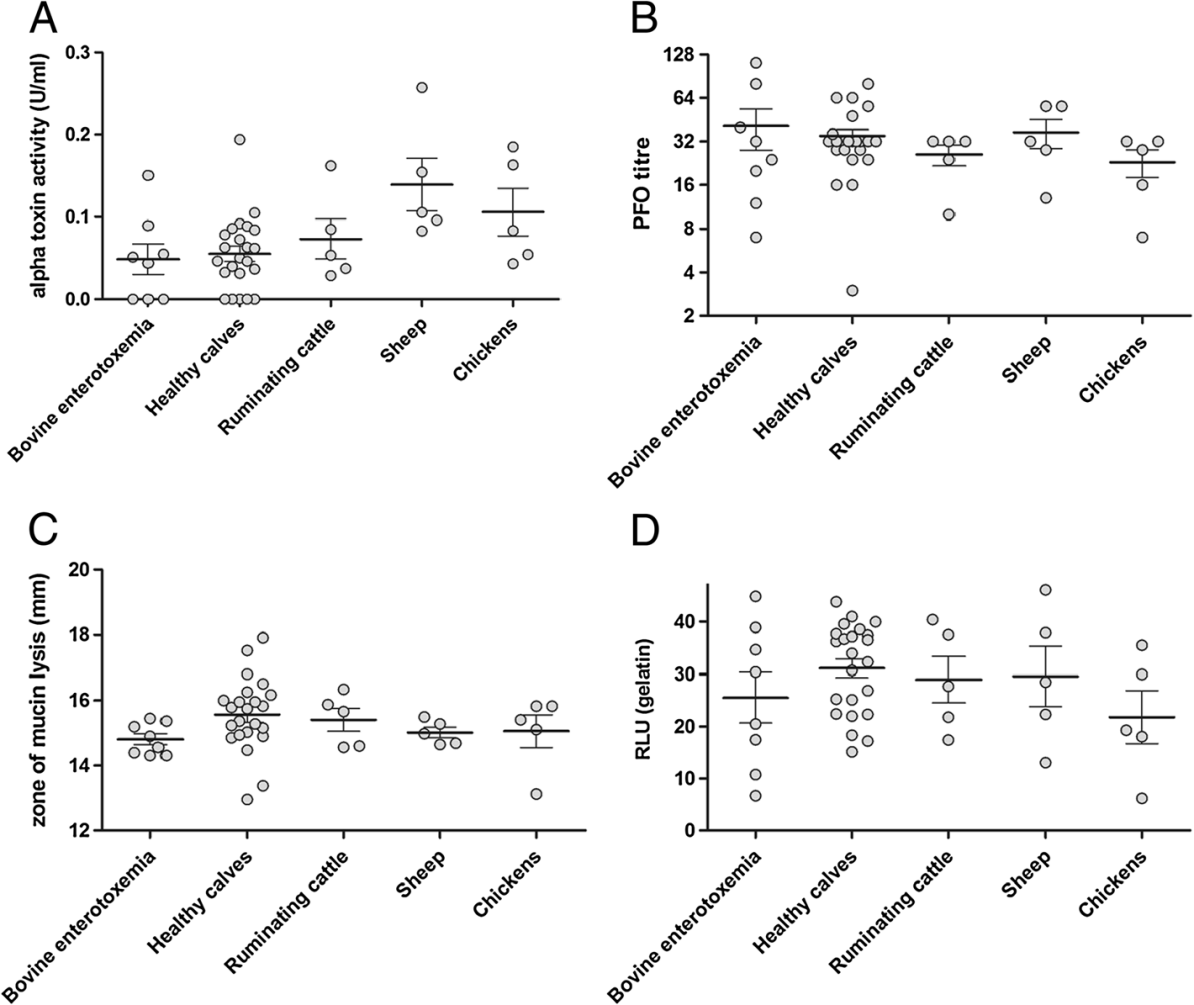
**Quantification of putative virulence factor activities of *C. perfringens *strains derived from cattle, sheep and chickens.** The lines represent the mean with the standard error of the means. **(A)** The alpha toxin activity in the supernatant of anaerobically grown *C. perfringens* strains was determined by measuring the lecithinase activity in an egg yolk agar well diffusion assay. **(B)** The perfringolysin O activity in the supernatant of anaerobically grown *C. perfringens* strains was determined by measuring the hemolysis of horse erythrocytes. The PFO titer is the reciprocal of the last dilution which showed complete hemolysis. Each difference in titer of one unit represents a twofold difference in perfringolysin O activity. **(C)** The mucinolytic activity of *C. perfringens* strains was assayed by adding cultures of strains to wells in TSA-mucin plates and quantification of zones of mucin lysis (in mm). **(D)** The potential to degrade the extracellular matrix was examined by measuring the breakdown of fluorescent labeled gelatin by supernatant of anaerobically grown *C. perfringens* strains. RLU = relative light units.

To determine whether alpha toxin and perfringolysin O levels differ between bovine enterotoxemia strains and strains from other sources, the culture supernatants of various type A strains were tested (Figure 1A). Lecithin breakdown was used as a measure of alpha toxin activity. The supernatants of the different strains showed high variability in alpha toxin activity, independent of their origin. In eight strains, the alpha toxin activity was below the detection limit of 15.6 10^-3^ U/ml (three strains originating from enterotoxemia cases and five strains from healthy calves). Perfringolysin O activity was determined by measuring the hemolysis of horse erythrocytes. Supernatants of strains originating from bovine enterotoxemia cases showed a high variability in hemolytic activity. The mean erythrocyte hydrolysis by strains from bovine enterotoxemia was not significantly different from other strains, independent of the origin (Figure 1B). For both alpha toxin and perfringolysin O, no significant differences between enterotoxemia strains and other *C. perfringens* strains could be observed.

### Mucinolytic and gelatinolytic activity is not higher in *Clostridium perfringens* strains from bovine enterotoxemia cases

The thickness of the mucin layer reflects an equilibrium between synthesis by the host and bacterial degradation by the intestinal microbiota. We tested the mucinolytic activity of *C. perfringens* strains from enterotoxemia cases and from other sources to evaluate whether enterotoxemia strains have higher potential to degrade the protective mucus layer. All strains exhibited similar mucinolytic activity (Figure 1C). To elucidate whether *C. perfringens* proteases might have the potential to contribute to the pathology of enterotoxemia, gelatinolytic activity was investigated as a measure for degrading extracellular matrix components within the gut. Gelatin was used as a specific proteolytic substrate to screen for clostridial protease activity that might contribute to intestinal host matrix degradation. A high variability of gelatinolytic activity was seen. The mean gelatin breakdown by strains from bovine enterotoxemia was not different from other strains, independent of the origin (Figure 1D). No significant difference between the groups could be observed.

### Enterotoxemia strains have no increased intra-species growth-inhibitory activity

**Figure 2:**
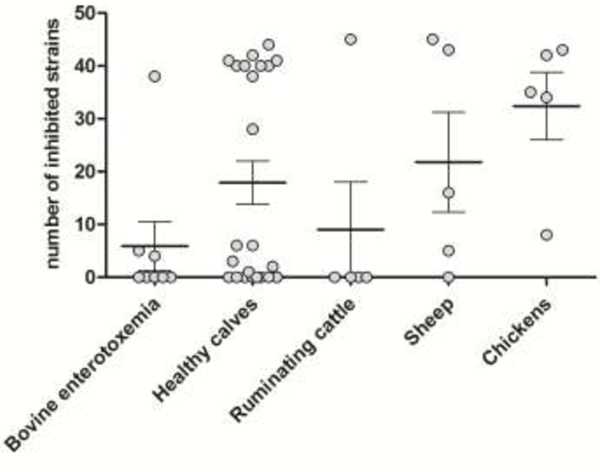
**Intra-species growth-inhibition.** Number of *C. perfringens* strains (N = 46) inhibited by the individual strains originating from bovine enterotoxemia cases, healthy calves, ruminating cattle, sheep and chickens. The lines represent the mean with the standard error of the means.

The intra-species growth-inhibitory activity is shown in Figure 2. Of all tested strains, sixty-one percent was able to inhibit growth of at least one other *C. perfringens* strain. There was a lot of variation between the inhibitory spectra of the tested strains. Some strains inhibited only a limited number of strains, while others had a broad inhibitory spectrum. Five out of eight enterotoxemia strains, eight out of twenty-three strains from healthy calves, four out of five ruminating bovine strains and one out of five sheep strains were unable to inhibit growth of any other strain. No significant differences between the groups could be observed.

## Discussion

*Clostridium perfringens* type A strains isolated from bovine enterotoxemia cases showed no difference in *in vitro* expression of a selection of potential virulence factors compared to strains from healthy cattle as well as various other animal species. This is in accordance with the capacity of lesion induction in a calf intestinal loop model, in which all tested strains are capable of inducing necro-hemorrhagic intestinal lesions, independent of the origin of the isolate [10].

No increased activity of the alpha toxin and perfringolysin O was detected in bovine enterotoxemia strains. In a recent study carried out with alpha toxin and perfringolysin O mutants we demonstrated an essential role of both toxins in bovine enterotoxemia [8]. The alpha toxin is the most toxic enzyme produced by *C. perfringens* type A strains and hydrolyses two major constituents of the eukaryotic membrane (phosphatidylcholine and sphingomyelin) causing membrane disruption and cell lysis [11,12]. In sublytic concentrations, alpha toxin leads to activation of signal transduction pathways and uncontrolled production of intracellular mediators [12-14]. Perfringolysin O is a pore-forming cytolysin which has the ability to kill eukaryotic cells by punching holes in their membranes [15]. As shown *in vitro* and in a calf intestinal loop model, alpha toxin and perfringolysin O seem to have a synergistic action in bovine enterotoxemia [8]. Considering this essential role of alpha toxin and perfringolysin O in bovine enterotoxemia, *C. perfringens* type A strains with higher activity of these toxins might be more virulent. Despite the importance of alpha toxin and perfringolysin O in enterotoxemia, strains originating from diseased animals showed no higher activities of these toxins *in vitro*.

No increased collagen degrading and mucinolytic potential was detected in strains from enterotoxemia cases. Collagen is widely distributed throughout the body and is an integral component of the connective tissue. Collagen disruption by bacterial collagenases may result in the loss of tissue integrity and subsequent tissue necrosis in the infected host and allow penetration of bacterial toxins to deeper tissues [16]. Therefore, the ability to produce collagenase may play an important role in the tissue destruction observed in bovine enterotoxemia. This *in vitro* study showed no difference in collagen destroying potential between *C. perfringens* strains from bovine enterotoxemia cases compared to strains from healthy cattle, sheep and chickens.

In addition to the collagen degradation, the mucinolytic activity of the *C. perfringens* strains may also contribute to the pathology of bovine enterotoxemia. The gastrointestinal tract represents a large surface of the host that interacts with the external world. A protective mucus layer covers the epithelial surface, forming a barrier between the lumen and the intestinal epithelium. It is a potential binding site for both commensal and pathogenic organisms [17,18]. *C. perfringens* type A strains with stronger mucinolytic activity may have an advantage in colonizing and degrading the protective mucus layer, which may lead to a compromised barrier function. Enterotoxemia strains, however were not different from the other strains with respect to their mucinolytic activity *in vitro*.

Another possible virulence trait that was explored is the ability to cause intra-species growth-inhibition. The potential of a pathogenic strain to suppress the growth of other *C. perfringens* strains has been shown in necrotic enteritis in broiler chickens, leading to single clone dominance in a broiler flock suffering from necrotic enteritis [9,19]. In contrast to the situation in broiler chickens, our results showed no difference in intra-species growth-inhibition between the enterotoxemia strains and other *C. perfringens* strains. In addition, a clonal population of *C. perfringens* does not seem to be essential for the development of bovine enterotoxemia as seen by RAPD (authors unpublished observations). Together this suggests that intra-species growth-inhibition probably does not play a role in the pathogenesis of bovine enterotoxemia.

None of the possible virulence traits examined in this study were distinctive for *C. perfringens* type A strains isolated from bovine enterotoxemia cases *in vitro*. This is in accordance with a recent observation from our laboratory that, when the conditions are favorable, strains from different origin are capable to provoke necro-hemorrhagic lesions in an intestinal loop model [10]. It cannot be ruled out that other possible virulence factors may be involved in disease. This was the case for necrotic enteritis in broiler chickens, an enteric disease caused by *C. perfringens* type A strains. The essential virulence factor for causing disease remained unclear until the NetB toxin, a previously unknown toxin specific for necrotic enteritis, was found [20]. Although the presence of such an undiscovered toxin in the pathology of bovine enterotoxemia cannot be excluded, it is unlikely because type A strains originating from bovine enterotoxemia cases as well as from healthy cattle and other animal species are capable of inducing lesions in an intestinal loop model [10].

## Conclusions

Strains from bovine enterotoxemia cases did not have a higher alpha toxin, perfringolysin O, mucinolytic or gelatinolytic activity in comparison to strains isolated from healthy cattle and other animal species. Also production of intra-species inhibitory substances was not higher in bovine enterotoxemia strains. This could indicate that yet another, hitherto unknown, *C. perfringens* virulence factor might be involved in the pathogenesis of calf enterotoxemia. Taking these results together with our previous observations that strains from various origin can induce the typical lesions in an intestinal loop model [10], it seems however more plausible that the primary trigger in bovine enterotoxemia is not *C. perfringens* and that *C. perfringens* is merely responsible for propagating and exacerbating the intestinal damage to the point that it becomes hemorrhagic and necrotic.

## Methods

### Bacterial strains and culture conditions

The 46 *C. perfringens* strains used in this study are listed in Table 1. One isolate per animal was used. Eight strains were isolated from enterotoxemic calves and 23 from healthy calves. Also five strains from ruminating cattle were included. In addition five strains isolated from chicken and five ovine strains were included. This study describes the *in vitro* characterization of *Clostridium perfringens* strains and thus does not need approval of an ethical committee.

**Table 1:** **Origins and toxinotypes of *C. perfringens *** **strains**

**Strain**	**Origin**	**Toxin genes**	**Reference**
**Bovine enterotoxemia**			
BCP62	BB calf, hemorrhagic gut	*cpa*^*+*^	[10]
BCP134	HF calf, hemorrhagic gut	*cpa*^*+*^	[10]
BCP256	BB calf, hemorrhagic gut	*cpa*^*+*^	[10]
BCP472	BB calf, hemorrhagic gut	*cpa*^*+*^	This study
BCP510	BB calf, hemorrhagic gut	*cpa*^*+*^	[10]
BCP544	BB calf, hemorrhagic gut	*cpa*^*+*^	[10]
BCP588	BB calf, hemorrhagic gut	*cpa*^*+*^	This study
BCP730	BB calf, hemorrhagic gut	*cpa*^*+*^	This study
**Healthy calves**			
BCP20	HF calf, abomasal ulcer	*cpa*^*+*^	[21]
BCP311	BB calf, rectal swab	*cpa*^*+*^	This study
BCP334	BB calf, rectal swab	*cpa*^*+*^	[10]
BCP447	BB calf, healthy gut	*cpa*^*+*^	[10]
BCP506	BB calf, rectal swab	*cpa*^*+*^	[10]
BCP513	BB calf, rectal swab	*cpa*^*+*^	This study
BCP740	HF calf, healthy gut	*cpa*^*+*^	This study
BCP747	BB calf, healthy gut	*cpa*^*+*^	This study
BCP795	BB calf, rectal swab	*cpa*^*+*^	This study
BCP796	BB calf, rectal swab	*cpa*^*+*^	This study
BCP797	HF calf, rectal swab	*cpa*^*+*^	This study
BCP799	HF calf, rectal swab	*cpa*^*+*^	This study
BCP806	BB calf, rectal swab	*cpa*^*+*^	This study
BCP808	BB calf, rectal swab	*cpa*^*+*^	This study
BCP812	BB calf, rectal swab	*cpa*^*+*^	This study
BCP821	BB calf, rectal swab	*cpa*^*+*^	This study
BCP822	HF calf, rectal swab	*cpa*^*+*^	This study
BCP823	HF calf, rectal swab	*cpa*^*+*^	This study
BCP824	HF calf, rectal swab	*cpa*^*+*^	This study
BCP825	HF calf, rectal swab	*cpa*^*+*^	This study
BCP828	BB calf, rectal swab	*cpa*^*+*^	This study
BCP836	HF calf, rectal swab	*cpa*^*+*^	This study
BCP837	HF calf, rectal swab	*cpa*^*+*^	This study
**Ruminating cattle**			
BCP783	HF bull, rectal swab	*cpa*^*+*^	This study
BCP815	BB cow, rectal swab	*cpa*^*+*^	This study
BCP820	BB cow, rectal swab	*cpa*^*+*^	This study
L2660	HF cow, rectal swab	*cpa*^*+*^	This study
L2664	BB cow	*cpa*^*+*^	This study
**Sheep**			
SCP1	Rectal swab	*cpa*^*+*^	This study
SCP2	Rectal swab	*cpa*^*+*^	This study
SCP3	Rectal swab	*cpa*^*+*^	This study
SCP4	Rectal swab	*cpa*^*+*^	This study
SCP5	Rectal swab	*cpa*^*+*^	This study
**Chicken**			
CP17	Healthy	*cpa*^*+*^	[19]
CP23	Healthy	*cpa*^*+*^*netB*^*+*^	[19]
CP24	Healthy	*cpa*^*+*^	[19]
CP56	Necrotic enteritis	*cpa*^*+*^*netB*^*+*^	[19]
NE18	Necrotic enteritis	*cpa*^*+*^*netB*^*+*^	[22]

BB = Belgian Blue, HF = Holstein Friesian.

Bacteria were isolated on Columbia agar (Oxoid, Basingstoke, UK) supplemented with 5% defibrinated sheep blood, 12 mg/l kanamycin sulphate and 30 000 IU/l polymixin B sulphate. The toxinotype of the *C. perfringens* strains was determined by a multiplex polymerase chain reaction (PCR), as described by Yoo et al. [23], while the presence of the enterotoxin, NetB and the consensus and atypical beta2 toxin genes were detected with previously described single PCR reactions [20,24,25]. The strains were cultured anaerobically for 24 h at 37°C in TGY broth (3% tryptone, 2% yeast extract, 0.1% glucose and 0.1% L-cysteine) for the toxin assays, in BHI broth (VWR, Leuven, Belgium) supplemented with 0.4% (w/v) glucose for growth-inhibition assays and measurements of gelatinolytic activity and in tryptone soy broth (TSB) (Oxoid) for quantification of the mucinolytic activity. Cell-free supernatants from the *C. perfringens* cultures were obtained by centrifugation followed by filtration of the supernatants through a 0.2 μm filter and stored at −20°C.

### Detection of toxin activity

To determine the alpha toxin activity in the supernatants, the lecithinase acitivity was assayed in an egg yolk agar well diffusion assay [26]. Therefore 7 mm diameter holes were punched out in Columbia agar (Oxoid) supplemented with 2% (vol/vol) egg yolk with the back of a 20-200 μl pipette tip and 20 μl of the tested supernatants was added to each hole. Pure alpha toxin (Sigma-Aldrich, St-Louis, USA) was used as standard. Plates were incubated at 37°C for 24 hours and scanned with a GS-800 calibrated densitometer (Bio-Rad Laboratories, Hercules, CA). The diameters of the zones of opacity were measured using Quantity One software (Bio-Rad Laboratories).

Perfringolysin O (PFO) activity in the supernatants was determined by measuring the hemolysis of horse erythrocytes using a doubling dilution assay as previously described [11]. The PFO titer is the reciprocal of the last dilution which showed complete hemolysis. The unit of activity was expressed on a logarithmic scale as a log2 value (titer), and consequently each difference in titer of one unit represents a twofold difference in perfringolysin O activity.

Both assays were performed in triplicate, with supernatants of two independent biological replicates of *C. perfringens* cultures grown in TGY.

### Detection and measurement of proteolytic activity

To determine the mucinolytic activity of the different *C. perfringens* strains, 7 mm diameter holes were punched out TSA plates supplemented with 0.5% (w/v) type II gastric mucin (Sigma-Aldrich) with the back of a 20-200 μl pipette tip. Twenty μl of each overnight culture was added to each hole (3 wells per overnight culture). The plates were anaerobically incubated for 16 hours at 37°C and subsequently stained for 30 minutes with amido black staining solution (Sigma-Aldrich). Plates were destained with destaining solution (25% isopropanol and 10% acetic acid) and scanned with a GS-800 calibrated densitometer (Bio-Rad Laboratories). Lysis of mucin was observed as a halo of clearing around the wells. The diameters of the zones of mucin lysis were measured using Quantity One software (Bio-Rad Laboratories).

Detection of gelatinolytic activity with an EnzChek Gelatinase/Collagenase Assay kit was carried out according to the recommendations of the manufacturer (Molecular Probes). Briefly, filter-sterilized supernatant of BHI overnight cultures was incubated with 12.5 μg/ml fluorescein-labeled gelatin substrate for 3 hours at 37°C (2 wells per overnight culture). Gelatinolytic activity was measured as an increase in fluorescence (excitation 495 nm, emission 515 nm) by a Fluoroskan Ascent Fluorometer and Luminometer (Thermo Fisher Scientific Inc., Waltham, USA). Both assays were performed with two independent biological replicates of *C. perfringens* cultures.

### *In vitro* growth-inhibition assay

All 46 *C. perfringens* strains were used in a checkerboard test for intra-species growth-inhibition as previously described [9]. Each strain was cultured anaerobically in BHI broth for 24 h at 37°C. The overnight cultures were diluted 1/1000 in 10 ml BHI agar and poured on the whole surface of BHI agar plates to obtain a bacterial lawn. With a sterile toothpick each *C. perfringens* isolate was transferred from the overnight culture to the agar plates seeded with the different *C. perfringens* strains. Absence of growth of the bacterial lawn around a colony results in an inhibition zone. After overnight incubation under anaerobic conditions, inhibition was evaluated. The test was performed in duplicate.

### Statistical analysis

All tests were performed in duplicate and data were analyzed using a Kruskal-Wallis test followed by a Dunn’s multiple comparison test. Statistical analyses were performed using GraphPad Prism 5 software.

## Competing interests

The authors declare that they have no competing interests.

## Authors’ contributions

EG, SV, LT, BV, BP, RD, PD and FVI participated in the design of the study. EG, performed the experiments and analyzed the data. BV collected the majority of *C. perfringens* strains. FH, PD, RD and FVI supervised the study. EG, FH, RD, PD and FVI wrote the paper. All authors contributed to the critical revision of the manuscript for important intellectual content and have seen and approved the final draft. All authors read and approved the final manuscript.
